# Long-Term Effect of Crop Succession Systems on Soil Chemical and Physical Attributes and Soybean Yield

**DOI:** 10.3390/plants13162217

**Published:** 2024-08-10

**Authors:** Milla S. S. Alves, Natanael M. Nascimento, Luiz Antonio F. M. Pereira, Thiago A. Barbosa, Claudio Hideo Martins da Costa, Tiara M. Guimarães, Aracy Camilla T. P. Bezerra, Deivid L. Machado

**Affiliations:** Campus Jatobá, Federal University of Jataí, Jataí 75804-020, Braziltiaraguimaraes@ufj.edu.br (T.M.G.); aracy.bezerra@ufj.edu.br (A.C.T.P.B.); deivid.machado@ufj.edu.br (D.L.M.)

**Keywords:** cover crops, crop–livestock integration, *Glycine max*, pearl millet, *Urochloa ruziziensis*

## Abstract

Most soybean producers in the Cerrado biome use the direct seeding system, making it essential to cultivate cash or cover crops in the off-season, to promote soil protection, as well as increase organic matter, which is directly related to improvements in the chemical and physical characteristics of these soils. In this sense, this work was conducted in Jataí, state of Goias, Brazil, to evaluate the physical-chemical attributes of the soil and the performance of soybeans cultivated in different crop succession systems cultivated for 6 years in the region of Jataí, GO. The experimental design was randomized blocks with four plots and four replications; the crops that followed soybeans were arranged as follows: T1—corn (*Zea mays*); T2—pearl millet (*Pennisetum glaucum*); T3—*Urochloa ruziziensis*; and T4—corn + *Urochloa ruziziensis*. Soybean yield components and grain yield were evaluated in two harvests (2020/2021 and 2021/2022). Deformed and undisturbed soil samples were collected in 2022 to assess soil fertility and for physical analysis. The data were subjected to analysis of variance (F test) and the means were compared using the Tukey test at 5% probability. The soybean–millet succession system stood out for the chemical and physical attributes of the soil: calcium, magnesium, base saturation, hydrogen + aluminum, and total porosity. The crop succession system did not affect yield for the two years analyzed, but the accumulated grain yields were higher in the crop succession soybean/corn intercropped. The results highlight the importance of using cover crops in improving the physical and chemical qualities of the soil in the long term. However, in the Cerrado, there is a predominance of the soybean/corn succession system motivated by financial issues to the detriment of the qualitative aspects of the soil, in which the introduction of *Urochloa ruziziensis* in intercropping with corn would improve the chemical attributes of the soil and have a long-term impact on the accumulated grain production.

## 1. Introduction

In the Cerrado Biome, from an edaphic point of view, the soils are classified as old, deep, flat, well-drained, and poor in fertility [1,2]. These regions are marked by two well-defined seasons with a rainy summer and a dry winter, which influence soil properties through weathering, whether physical, chemical, or biological [3]. 

The dynamics of soil structure, such as pore space, density, and stability of aggregates, in addition to nutrient dynamics, as well as the leaching and fixation of nutrients to clay colloids, are factors subject to various interferences, positive or negative, depending on the conditions to which the soil surface is exposed [4]. It is known that farming in tropical soils is a challenge from the point of view of soil quality because of the predominance of a high degree of weathering, low level of fertility, high acidity, and low organic matter (OM) content [5]. Under these conditions, cropping must be carried out with management to maximize the productive potential.

In Brazil, over 32 million hectares are cropped through the No-tillage system (NTS), where crop rotation, straw persistence, and minimal soil disturbance are the pillars of the success of this system [6]. Regarding soil conditions, crop rotation and the persistence of straw on the surface are determining factors in the choice of cover crops. The persistence of straw on the surface allows for a slower release of nutrients resulting from the decomposition of plant material, in addition to its influence on various physical properties. Organic matter promotes stability to aggregates, directly influencing soil structure, water infiltration, retention capacity, aeration, and root development, as well as nutrient availability and microbial activity [7].

The quality and quantity of residue produced by cover crops, as well as the persistence on the surface, influence the success of the NTS. In the Cerrado, the main challenge for establishing NTS is the difficulty in the production of straw, especially in non-irrigated conditions because of the small productivity of autumn–winter crops. Furthermore, climatic conditions do not favor the maintenance of plant residues on the soil surface, due to the accelerated decomposition to which they are exposed [8]. The systematic indication of cover crops associated with a crop-rotation system is the main strategy for achieving the accumulation of straw on the surface, in addition to increasing yield for the main crop. Hence, there is a preference for species that promote, simultaneously, the possibility of controlling weeds and providing mulch on the soil, promoting improvements in chemical, physical and biological attributes [9].

Because of its high C/N (carbon/nitrogen) ratio, which promotes greater permanence on the soil surface associated with large production of leaf and root mass, the grass family (Poaceae) is the ideal choice for the crops that precede soybean harvest. These plants are also chosen due to their aggressive root system, which helps in the structuring and aeration of the soil, in the absorption of nutrients from deeper layers and releases through their gradual decomposition, in the rational use of water, becoming an economically profitable source. Furthermore, the use of grasses, isolated, mixed, and intercropped with grains, can favor the diversification of crops sown as a second crop in production systems, resulting in greater production efficiency and quality biomass added to the soil [10].

Therefore, concerning these cover crops, used as organic matter, soil conditioners, and also as a source of commercialization, millet, brachiaria, and especially corn have stood out as off-season crops that also benefit the NTS through straw production.

In this context, the objective of this work was to evaluate the physicochemical attributes of the soil depending on production systems for soybean cultivation in the region of Jataí, state of Goias.

## 2. Results and Discussion

### 2.1. Soil Chemical Attributes

**Figure 1:**
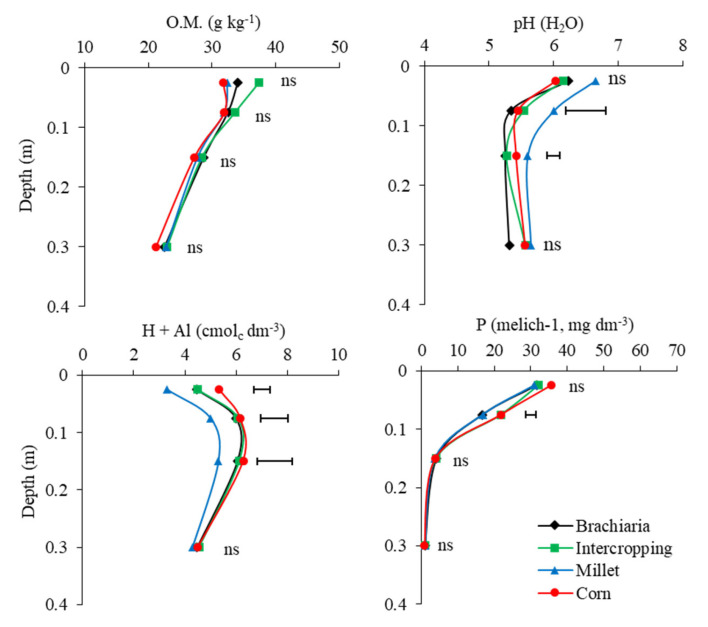
Soil organic Matter (OM) soil acidity (pH), potential acidity (H  +  Al) and phosphor (P) in layers 0.0–0.05, 0.05–0.10, 0.10–0.20 and 0.20–0.40 m depth, after soybean growing season 2021/2022 in function of the crop-production systems. Jataí, GO. Horizontal bars indicate the least significant difference of Tukey test (*p* < 0 .05) for each depth and “ns” indicates that the ANOVA was not significant.

**Figure 2:**
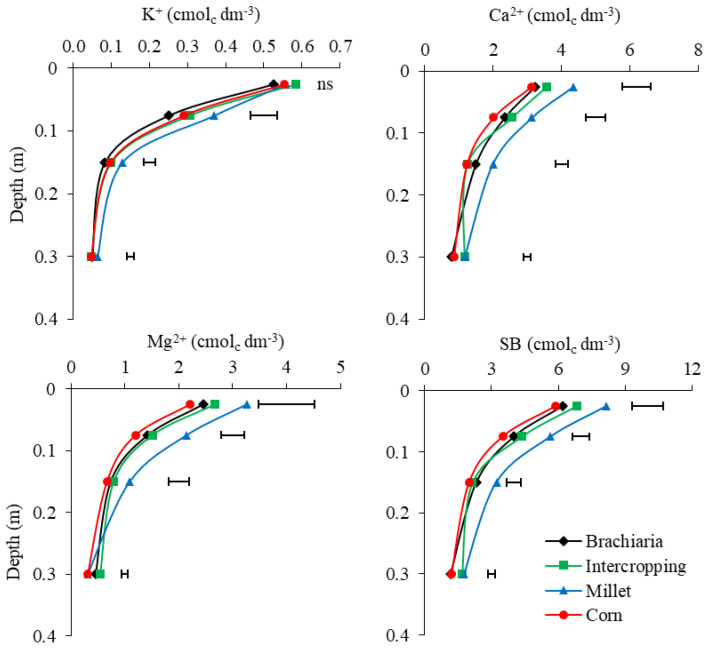
Potassium (K^+^), calcium (Ca^2+^), magnesium (Mg^2+^) and sum of bases (SB) in layers 0.0–0.05, 0.05–0.10, 0.10–0.20 and 0.20–0.40 m depth, after soybean growing season 2021/2022 as a function of the crop-production systems. Jataí, GO. Horizontal bars indicate the least significant difference of Tukey test (*p* < 0.05) for each depth and “ns” indicates that the ANOVA was not significant.

**Figure 3:**
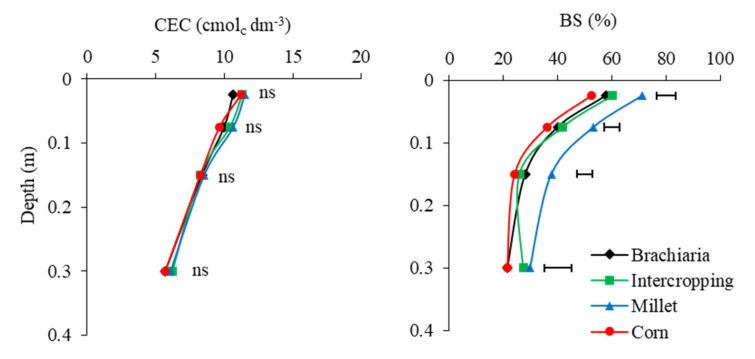
Cation exchange capacity (CEC) and base saturation (BS) in layers 0.0–0.05, 0.05–0.10, 0.10–0.20 and 0.20–0.40 m depth, after soybean growing season 2021/2022 in function of the crop-production systems. Jataí, GO. Horizontal bars indicate the least significant difference of Tukey test (*p* < 0.05) for each depth.

Data on soil chemical attributes are shown in Figure 1, Figure 2 and Figure 3. The adopted succession systems did not alter the soil organic matter content at any of the depths evaluated, which is corroborated by the CEC, which is influenced by soil organic matter. Ref. [11] observed an increase in total organic carbon content in crop–livestock integration systems after 8 years, working with different plant families. However, in the systems evaluated, the crop successions adopted were legumes (soybean) in spring/summer and grasses (corn, millet and brachiaria) in autumn/winter without alternating families in the same growing season. Thus, grasses are efficient in carbon accumulation due to the substantial and extensive root system, which associated with legumes has great potential to fix atmospheric carbon into more recalcitrant compounds, thus contributing to the success of carbon sequestration [12].

**Figure 4:**
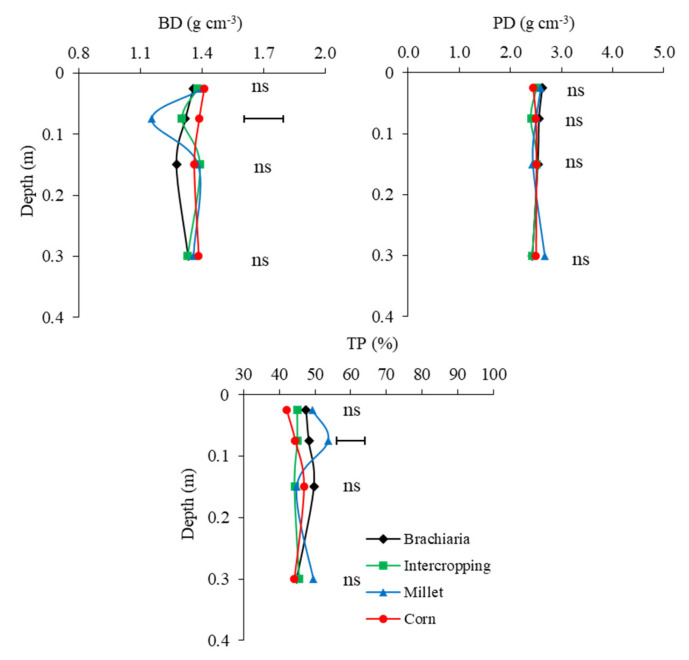
Bulk density (BK), particle density (PD), total porosity (TP) in layers 0.0–0.05, 0.05–0.10, 0.10–0.20 and 0.20–0.40 m depth, after soybean growing season 2021/2022 as a function of the crop-production systems. Jataí, GO. Horizontal bars indicate the least significant difference of Tukey test (*p* < 0.05) for each depth and “ns” indicates that the ANOVA was not significant.

The pH values were higher in the 0.05–0.10 and 0.10–0.20 m layers in the soybean/millet succession system, reflecting the lower potential acidity (H + Al) in the same layers. Although millet is a species with a lower C/N ratio compared to other species, which could have the capacity to generate greater soil acidity due to residue hydrolysis reactions that release H^+^ ions [13,14], the factors mentioned above and the increase in porosity observed at a depth of 0.05–0.10 m (Figure 4) may have promoted greater movement of the lime applied to the surface, attenuating the acidity of the soil.

The greater acidity in the soybean/corn and soybean/corn intercropped succession systems may be related to the nitrogen fertilization carried out on the corn crop (Appendix A, Table A1), which is considered the main factor in the acidification of agricultural lands [15]. The soybean/brachiaria succession system with the cations immobilized in the brachiaria plants led to a net production of H^+^ production by roots to maintain the charge balance, particularly contributing momentarily to the acidification [16], since the soil was collected while the plants were still alive.

The soil P levels were higher in the succession system soybean/corn and soybean/corn intercropped in the 0.05–0.10 m layer (Figure 1). This occurs due to the fact that these systems include the addition of phosphate fertilizers in the corn base fertilizer (Appendix A, Table A1), and due to the low mobility of this nutrient in the soil because of high fixation in highly weathered soils, causing great competition between the soil and plants with the applied fertilizer, with a large part of the P retained in the mineral fraction of the soil via high-energy bonds [17,18], restricting the effects of phosphate fertilizers to the layers closest to the application site.

However, Ref. [10] found an increase in P content in areas cropped with brachiaria in a Cerrado area. These effects may also occur in the long term, especially in layers with less influence of phosphate fertilization (0.10–0.20 and 0.20–0.40 m), where it is already possible to notice a trend towards increasing P contents, with values of 4.13 and 3.99 mg dm^−3^ in the 0.10–0.20 layer and 0.98 and 0.91 mg dm^−3^ in the 0.20–0.40 m layer, in the soybean/brachiaria and soybean/corn intercropped systems, respectively, which were 8% and 29% higher than the soybean system soybean/corn, in the layers 0.10–0.20 and 0.20–0.40 m, respectively.

As for K, levels were higher in soybean/millet succession systems in layers 0.05–0.10 and 0.10–0.20 m (Figure 2). Ref. [19] observed greater accumulation and release with the cultivation of millet cover crops in a Cerrado area. According to [20], plants with greater phytomass accumulation can capture more nutrients and, with a large part of the biomass, return them to the soil. Concerning millet straw, due to its short cycle, when it matures, the degradation process begins, which quickly releases K back into the system [21,22].

The Ca and Mg showed significant differences for crop succession systems, with soybean/millet being the system with the most positive influence in all layers, with average concentrations in the layers of 2.67 and 1.75 cmol_c_.dm^−3^, respectively, and soybean/corn being the system that showed the lowest averages of 1.81 and 1.09 cmol_c_.dm^−3^ (Figure 2). Ref. [23] explains that the deep root system of millet allows the absorption of the nutrient in subsurface layers, a fact that may explain these higher surface concentrations after the decomposition of millet residues.

A similar result was observed by [24], taking into account, however, the nutritional requirements of millet concerning nutrients and that 100% of the grass remained in the soil, even after physiological maturation. Ref. [25], when evaluating the accumulation of nutrients in grasses, observed that the highest export and foliar concentration of Ca and Mg was observed for millet. Millet can extract 32 kg ha^−1^ of Ca and 21 kg ha^−1^ of Mg and accumulate 5 t.ha^−1^ of dry matter mass [26]. In this case, the accumulation of nutrients in the aerial part of plants can be associated with the highest concentrations in the most superficial layer, resulting from the decomposition of plant material and cycling.

However, corn, which is commonly used as a second crop in the southwest region of Goias, had the lowest Ca and Mg values compared to other treatments and at all depths, except the 0.20–0.40 m layers. This depletion may be related to the greater export of nutrients by the crop in grain production, as reported by [27], who observed the highest levels of the nutrient in grain export (9.20 t.ha^−1^) and the lowest in persistent straw (7.2 t.ha^−1^).

In the SB, the crop succession system soybean/millet promoted the greatest increase at all depths (Figure 2). At depths within each crop succession system, SB was greater in the most superficial layer (0.00–0.05 m), with the highest values being 8.16 cmol_c_ dm^−3^. It is important to highlight that the sum of exchangeable bases (Ca^2+^, Mg^2+^ and K^+^) is indicative of the soil quality. In works with an intercropping of oats and lupine, Ref. [28] observed increases in SB in more superficial layers, which promoted the productivity of successor crops. According to [29], grass straws provide nutrients to successor crops, in the medium and long term, especially in the surface layer. This fact is also shown by [30], where the results confirm the prominence of millet as a supplier of nutrients for commercial successor crops when compared to cover crops with leguminous species.

It is known that the increase in SB occurs as the base levels are high. In this work, the higher concentrations of K, Ca and Mg for millet explain the higher levels for the crop succession system soybean/millet treatment in all layers. Ref. [31] observed an increase in Ca, Mg and K in the 0.00–0.10 m layer in different types of grasses after three years of cultivation.

The crop succession system soybean/corn showed the lowest SB values, ranging from 1.21 cmol_c_.dm^−3^ to 5.59 cmol_c_.dm^−3^, decreasing according to depth (Figure 2). These lower values are attributed to the export of Ca, Mg, and K in the order of 3.6, 42, and 10.8 kg ha^−1^, respectively, where a large part is removed from the area at grain harvesting, in which the remainder is the nutrients returned as waste decomposes. However, even the corn crop exporting large amounts of nutrients still returns substantial amounts of nutrients to the surface layers of the soil, linked to the persistence of straw and winter conditions in the off-season, with lower temperatures and reduced microbial activity in the decomposition of residues [32,33,34].

As a result, it can be seen that the intercropping crop succession system becomes an alternative for producers who practice the crop succession soybean/corn. For the nutrient contents analyzed so far (Figure 1 and Figure 2), this treatment was the second most prominent, which leads to the inference that intercropping becomes an excellent choice for integrated cash crops and the benefits of cover crops, for improving nutrient levels for the next harvest, in addition to greater soil protection over the year [35,36]. The use of brachiaria to form straw has become a viable alternative for maintaining NTS, as it produces a high amount of dry matter [37] and returns nutrients to the soil, and corn is an excellent profitable source in the off-season.

The distribution, content and, consequently, the availability of bases undergo changes, mainly due to the CEC of the soil and amounts of organic matter in the soil [38]. However, for CEC levels there were no differences among treatments (Figure 3). According to [39,40], the effects of using cover crops on soil fertility include the addition of nutrients, higher CEC and lower acidity. Ref. [41] found in Oxisol that crops with cover crops showed greater accumulation of K, Ca and Mg and increased soil pH and CEC. Concerning the grasses investigated by [42], millet was the production system that best increased CEC levels, followed by brachiaria and sunn hemp, respectively.

For V%, the crop succession system soybean/millet showed the highest value for the variable, with mean values of 48%, which are approximately 15% more compared to the values found for corn. These results reflect those observed for the levels of H + Al, K, Ca and Mg in the soil (Figure 1 and Figure 2). Knowledge of the V% level says a lot about the fertility of the soil, as it indicates how many of the colloids are occupied by exchangeable bases and that the lower this value is, the greater the adsorption of H^+^ and Al^3+^ and the lower that of the SB elements, with an increase in the toxic effect on the root area, therefore reducing yield. Ref. [43], studying different cover crops and the possible effects on chemical attributes, found that millet (ADR 300), grain sorghum (*Sorgum bicolor* cv BRS 307), jack beans (*Canavalia eusiformes*) and crotalaria juncea (*Crotalaria juncea*) in NTS were the crops that improved, in the 0.00–0.10 m layer, the values of SB, P and V% for the successor crops.

### 2.2. Soil Physical Attributes

**Figure 5:**
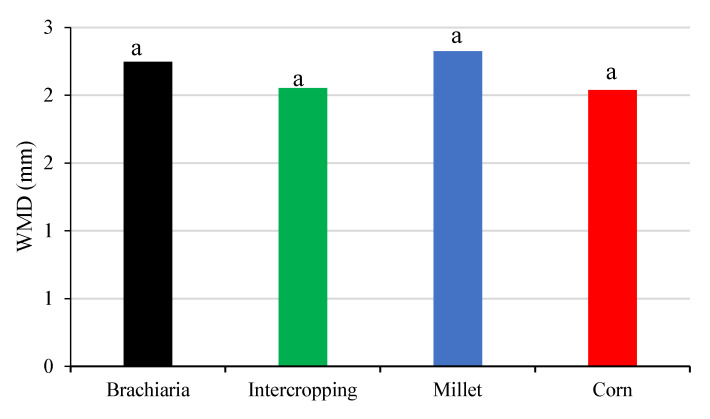
Weighted mean diameter (WMD) in layer 0.0–0.20 m, after soybean growing season 2021/2022 as a function of the crop-production systems. Jataí, GO. Different letters represent significative difference between the treatments.

The results of the physical properties of the soil are shown in Figure 4 and Figure 5. PD and WMD did not show significant differences between the crop succession systems, while BD and TP for crop succession systems were statistically different at depth 0.05–0.10 m.

The crop succession soybean/millet presented the lowest BD values and highest TP values in the layer 0.05–0.10 m. Millet has been chosen as a plant with the ability to break through compacted layers due to its rusticity, resistance to drought, and adaptation to different soil and climate conditions, which allow its development in adverse conditions [35]. The surface layers of the soil are generally provided by root residues, which are responsible for forming biopores and promoting the structural conditions of the soil [44]. The close relationship between soil porosity and root growth was observed by the authors who found that the greater the root growth, the greater the increase and continuity of pores [45,46]. The results of this work are similar to those discussed by [47], that of all the grasses observed, corn was not the most efficient for the soil porosity effect. But they are in line with several authors, who had corn as the prominent grass, whether grown in intercropping or alone, in improving the physical attributes of the soil, particularly for BD and TP.

Refs. [48,49] showed that with the rise in OM content in the surface layer, the BD tend to decrease, improving the structural quality of the soil and promoting an increase in TP. Corroborating these results, Ref. [45] concluded a better performance among cover crops for the millet variety ADR300 at BD and TP values of 1.65 g.cm^−3^ and 51%, respectively, in LVdf in southwestern Goias.

The Ds values indicate the condition of the structural conservation, and influence attributes such as water infiltration and retention in the soil and root development of plants [50,51]. The value found in this work for the Ds variable was 1.34 g.cm^−3^. Under natural conditions, Ref. [52] indicates Ds below 1.4 g.cm^−3^ for soil without compaction characteristics; therefore, the values in this work are consistent with the uncompacted structure.

On the other hand, BD depends on the organic and mineral fraction of soil. The values reported in the literature for this variable are close to 2.65 g.cm^−3^ for soils rich in minerals and 0.9 to 1.3 g.cm^−3^ for soils rich in OM [53]. The analyzed results presented an overall mean of 2.51 g.cm^−3^ (Figure 4), highlighting that the soil in question has high levels of iron oxides (LVdf), which explains the fact that even after the seven years of addition of organic material to which this soil has been subjected, the values are closer to those established for the mineralogical composition, showing that the variable is independent of the type of management adopted, explaining the non-significance for the crop succession systems.

TP is the characteristic that determines the portion of empty spaces in the soil, which corresponds to the space where the dynamic processes of air and soil solution occur, which is fundamental for the development of crops [54]. The overall mean of 46.56% of total soil porosity (TP) is close to the value considered ideal by [55], which is 50% of porosity. Ref. [56] found similar values in clayey soils for different grasses grown under cover, from 50 to 52% of TP in the 0.00–0.10 m layer of the soil. These values are higher than those found in this work, which was 44.43 to 49.31%, where crop succession soybean/millet is the treatment with the greatest increase in pores (49.31%) and the crop succession soybean/corn was the smallest (44.43%).

Values of the weighted mean diameter (WMD) were not significant among the crop succession systems evaluated (Figure 5). The analysis of the aggregates allows us to infer the structure of the soil, that is, how the particles are organized. For the different grasses in coverage, the aggregates presented sizes of 2.18 to 3.13 mm in the 0.00–0.10 m layer, and the results were attributed to the higher concentration of OM due to the cementing effect of organic particles [57]. For the formation of the aggregate, it is necessary that the colloids in the soil are flocculated and that all components of the aggregate are subsequently stabilized by some cementing agent [58], where the primary ones are the humic substances, silicate clays and oxides of iron and aluminum [59]. The stability of soil aggregates tend to increase more in soils under grasses than in soils under legumes, as observed by [60].

The results known for soil physical attributes in several experiments are observed over a long period and have shown the beneficial effects of the accumulation of organic residues on the soil surface through the NTS on properties such as BD, TP and WMD [61,62]. Therefore, as there were no changes in organic matter levels until 2022, the effects on the soil physical properties were restricted to the growth of the species root system, but this work highlights the importance of long-term experiments, in which the integration of cash crops with cover plants may bring greater benefits in the long term.

### 2.3. Vegetative and Productive Components of Soybean

**Figure 6:**
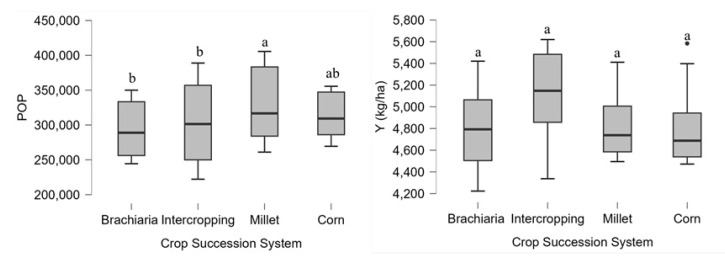
Plant final population (POP) and Yield (Y) as a function of crop succession systems, averages for growing season 2020/2021 and 2021/2022. Jataí, GO. Different letters represent significative differences between the treatments.

The soybean yield components and grain yield and the analysis of variance are shown in Table 1. The number of pods (NP), the number of grains per pod (NGP), the number of total grains (NG), the mass of 400 grains (M400), plant population (POP) and final yield (Y) did not change the interaction between the factors. However, the crop succession systems influenced the sources of variation final POP and Y (Figure 6), and the growing season factor influenced the NP, POP and Y. The means found for NP were 38.95 pods per plant and 97.20 grains per plant, and 400-grain mass was 62.95 g. The growing season 2021/2022 had the highest values of NGP and Y.

The plant population of soybean was lower when cultivated after brachiaria or intercropped corn (Figure 6). This occurred due to the high amount of straw in these systems, which tends to increase the “enveloping” of the seeds; that is, they remain between the soil and the straw, reducing the chances of germination.

Grain yield, despite the significant difference in ANOVA, was similar in the crop succession systems (Figure 6), but when we analyzed the accumulated yield of the two soybean-growing season, the soybean/corn intercropped succession system provided the highest grain yield. These results show that even though there is no significant difference in the agricultural year, the benefits of these succession systems seen in the medium to long term must consider the productivity accumulated during the period. These systems intercropped with forage grasses can significantly increase, in some cases, soil organic matter and soil cation exchange capacity in a short period of time [37,63].

To achieve high yield of soybean cultivars, it is necessary to have a balanced interaction between the plant, management and the production environment. High yields are achieved when environmental conditions are favorable at all growth stages [64]. Therefore, such facts associated with the availability of nutrients, the persistence of the straw and the genetic quality of the seeds may have influenced these differences among the productive components [65,66].

## 3. Materials and Methods

### 3.1. Experimental Area Characterization

The work was conducted in the experimental area of the School Farm of the Federal University of Jataí (UFJ), located in the municipality of Jataí, state of Goias (GO), within the geographic coordinates of 17°52′53″ S and 51°42′52″ W and 685 m above sea level, located in the central-west region of Brazil. According to the Köppen classification, the region’s climate is Aw, a tropical savannah with defined dry and rainy seasons, with an average annual temperature of 23.7 °C and a rainfall rate of 1800 mm.year^−1^ [67].

The experiment was conducted in a dystroferric Red Latosol (LVdf) with a clayey texture [68], whose chemical characterization before the implementation (2016) is shown in Table 2. In 2020, soil samples were collected in all systems at a depth of 0.00–0.20 m in all treatments to guide the fertilization of soybean crops (Table 3).

### 3.2. Experimental Design and Treatments

The experimental design used in this work was the randomized blocks, with four treatments and four replications. The treatments consisted of four agricultural succession systems: T1—soybeans in the harvest and single corn (*Zea mays*) in the second harvest (considered as a control); T2—soybeans in harvest and millet (*Pennisetum glaucum*) in second harvest; T3—soybeans in the harvest and brachiaria (*Urochloa ruziziensis*) in the second harvest; and T4—soybeans in the harvest and in the second harvest corn intercropped with brachiaria).

Cover crop treatments were distributed in monoculture with a spacing of 0.45 m and, in intercropping, corn in the row and brachiaria between the rows. The experimental area was 1080 m^2^ (60 × 18 m), with each experimental unit measuring 67.5 m^2^ (15 × 4.5 m). Cover crops were chosen by considering the common crops in the region.

### 3.3. Experiment Conduction

**Figure 7:**
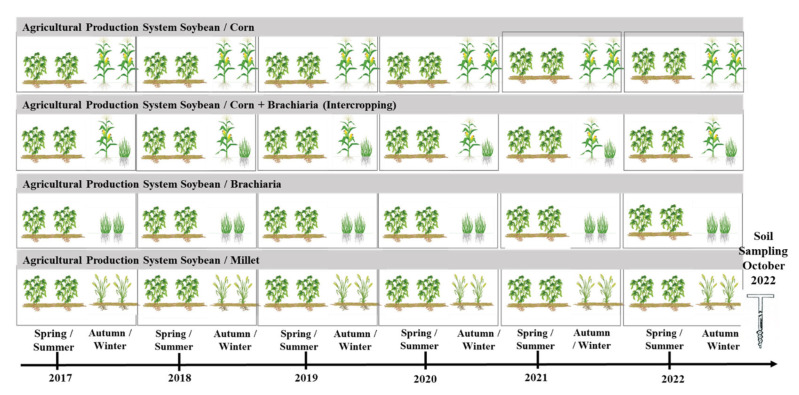
Schematic of crop succession systems and experiment timeline.

The area has been cropped since October 2016 with different production systems, which recommend direct sowing and soybeans as the main crop, resulting in six soybean harvests and five off-season plantings with cover crops (Figure 7).

This experiment was conducted on two soybean-growing seasons, 2020/2021 and 2021/2022. Soybean sowing throughout all experimental years (2016–2022) was carried out in October, at a spacing of 0.45 m and using cultivars recommended for the region, and crops sown in autumn/winter were also sown at a spacing of 0.45 m (Appendix A, Table A1). In the 2020/2021 and 2021/2022 harvest, the cultivar used was Foco Ipro BRASMAX^®^ (Uniggel, Jataí, Brazil), which has the following agronomic characteristics: indeterminate growth habit, poor branching and a thousand-grain mass of 176 g.

Insect pests and diseases were tracked over the cycle and phytosanitary management was carried out according to the needs of the crop (Appendix A, Table A2). The product applications were carried out with the aid of a “carriola sprayer”, without the introduction of machines into the area in order to minimize any impact on the soil surface, highlighting that only at planting and harvesting were agricultural machinery used in the experimental area.

The 2020/2021 crop was sown on 23 October 2020 and harvested on 22, 23 and 24 February 2021; for the 2021/2022 season, soybean was sown on 23 October 2021 and harvested on 23 and 24 February 2022. For morphological and yield analyses, samples were collected in both crops, with 10 plants in the central lines, discarding 1 meter from the ends as borders. Mechanical threshing was carried out shortly afterward, close to the experimental field.

After harvesting the 2020/2021 and 2021/2022 crops, the area was desiccated for subsequent sowing plants in autumn/winter with the application of 3.5 L ha^−1^ of the product Glyphosate. Crops sown in autumn/winter, in all treatments, were sown mechanically in March of the two years of the experiment, 2021 and 2022. The corn (single and intercropped) used in the experiment was the one recommended for the region, and the millet and the forage were ADR300 variety and *Urochloa ruziziensis*, respectively (Appendix A, Table A1). At the end of the cover crop cycle, only corn cobs were harvested, while the other crops entered natural senescence and remained in the area.

### 3.4. Soybean Yield

For the productive components, the number of pods (NP), number of grains per pod (NGP), mass of 400 grains (M400), final population (POP) and yield (Y) were evaluated.

Before the soybean harvest, in both harvests, the final population was set using a tape measure in the useful area of the plots, delimiting two central lines of two meters in length, and subsequently, the mean was defined. After harvesting, 10 plants were separated to evaluate NP and NGP through manual counting. Then, M400 was obtained, using an electronic seed counter and precision scale, where moisture was corrected to 13% and subsequently the average was in grams (g).

After all evaluations, yield was determined with the plants in the useful area of each plot, subjected to mechanical threshing and removing all impurities from the grains. The harvested material was weighed using an analytical scale; the moisture was corrected to 13% moisture.

### 3.5. Soil Evaluations

Soil samples were collected 60 days after the off-season corn harvest, on 10 October 2022. Trenches 40 cm in depth were opened in each of the 16 plots. The deformed and undeformed samples were collected at the following depths: 0–5 cm, 5–10 cm, 10–20 cm, 20–40 cm, totaling 64 samples. For aggregate stability analyses, monoliths were collected in the 0–20 cm layer in each trench, with 16 monoliths being collected.

The deformed samples were used in chemical analyses, according to the methodology described in [69]. The macronutrients P and K were extracted with Mehlich solution^−1^, with reading through spectrophotometry for P and flame spectrophotometry for K, Ca, Mg and Al were extracted using KCl solution 1 mol.L^−1^ and reading with the aid of atomic absorption. Soil organic matter (SOM) was determined through wet oxidation with K dichromate in a sulfuric medium and determined using titration.

The undisturbed samples were collected using volumetric rings, 5.7 cm in diameter and 6.3 cm in height, and the monoliths were placed in plastic bags, properly identified and sent to the laboratory. For execution of the analyses of undisturbed samples, initially, the excess soil was removed from the ends of the rings to standardize the samples for mass and volume determinations.

Soil bulk density (Ds) was determined using the volumetric ring method, particle density (Dp) using the volumetric flask method and total porosity (Pt) using the indirect method, following the methodology proposed by [69]. Aggregate stability (AS) was defined according to the methodology described by [70], using the weighted mean diameter (WMD) in the wet process.

### 3.6. Statistical Analysis

Data of the soil physical and chemical attributes were subjected to analysis of variance (ANOVA) and the means were compared separately by depth with the Tukey test at 5% probability using the Sisvar 4.2 statistical program [71]. Data of soybean production parameters were subjected to ANOVA—repeated measures, considering growing season as repeated measures, using mixed models with the JASP 0.19 software (JASP Team, Amsterdam, The Netherlands, 2024). 

## 4. Conclusions

The soybean/millet succession system improves the chemical characteristics of the soil. However, in Cerrado the soybean/corn succession (control) predominates, which among the systems evaluated presents the lowest nutrient content in the soil, and the simple addition of brachiaria in the intercropped soybean/corn succession systems improves the chemical quality of the soil. Soil density and soil porosity improve in the soybean/millet succession system at a depth of 0.05–0.10 m, but soil physical parameters are less sensitive in the short/medium term, indicating the need to continue the present work. Chemical and physical changes were not sufficient enough to alter the productive components and productivity of soybeans in the growing season, but the grain yield accumulated showed the crop succession system soybean/corn intercropped was an alternative to maximize land use by improving chemical parameters in relation to the traditional system soybean/corn.

## Figures and Tables

**Table 1:** Summary of the analysis of variance (F probability) and soybean yield components and grain yield as a function of crop succession systems.

Crop Succession Systems	NP (no.)	NGP (no.)	NG (no.)	M400 (g)	POP (no.)	Y (kg.ha^−1^)	∑Y (kg.ha^−1^)
Corn	36.35	2.49	90.23	62.38	313,771	4835	9669 b
Millet	38.00	2.45	93.01	61.86	329,512	4835	9669 b
Brachiaria	41.39	2.51	103.59	62.73	293,401	4811	9622 b
intercropping	40.06	2.54	101.98	64.83	303,123	5107	10,203 a
2020/2021	39.93	2.33	93.02	62.30	354,973	4576.5	-
2021/2022	37.98	2.67	101.38	63.60	264,931	5217.2	-
SV	NP (no.)	NGP (no.)	NG (no.)	M400 (g)	POP (no.)	Y (kg.ha^−1^)	∑Y (kg.ha^−1^)
Treatments (T)	0.154 ns	0.199 ns	0.098 ns	0.153 ns	0.018	0.034	0.069
Growing Season (GS)	0.232 ns	<0.001	0.058 ns	0.052 ns	<0.001	<0.001	-
T × GS	0.267 ns	0.20 ns	0.233 ns	0.065 ns	0.084 ns	0.896 ns	-

ns: Not significant; NP: Number of pods; NGP: Number of grains per pod; NG: Total number of grains; M400: 400-grain mass; POP: Plant final population; Y: Final yield; ∑Y: accumulated yield 2020/2021 and 2021/2022; SV: Source of variation. Means followed by the same letter are not statistically different from each other by the test of Tukey at 5% probability.

**Table 2:** Soil chemical and granulometric characterization (0–0.20 m) in the experimental area before installing the experiment. Jataí, GO, 2016.

pH	OM	Meh. P	H + Al	K	Ca	Mg	SB	CEC	V
H_2_O	g.kg^−1^	mg.dm^−3^	-------------------- cmolc.dm^−3^ ----------------------						%
5.10	36.0	16.1	5.75	0.21	2.76	1.02	3.99	9.74	40.96
		Clay			Sand				Silt
		----------------------------- g.kg^−1^ -----------------------------							
		585			240				175

**Table 3:** Soil chemical analysis collected at a depth of 0.00–0.20 m in 2020 for the purposes of fertilizing the systems.

pH	OM	Meh. P	H + Al	K	Ca	Mg	SB	CEC	V
H_2_O	g.kg^−1^	mg.dm^−3^	---------------------- cmolc.dm^−3^ ---------------------						%
**5.78**	31.8	21.4	4.09	0.26	2.92	1.55	4.73	8.82	46.3

pH: hydrogen potential in H_2_O; OM: organic matter; Meh.P.: Mehlich phosphorus; Al: aluminum; H: hydrogen; K: Mehlich potassium; Ca: calcium; Mg: magnesium; SB: sum of bases; CEC: cation exchange capacity; V%: base saturation.

## Data Availability

Data are contained within the article.

## Appendix A

**Table A1:** Cultivar, sowing density and fertilizer management carried out in the 2016/2017, 2017/2018, 2018/2019, 2019/2020 and 2020/2021 harvests in each crop.

		Growing Season									
		2017	2017/2018	2018	2018/2019	2019	2019/2020	2020	2020/2021	2021	2020/2021
		Corn	Soybean	Corn	Soybean	Corn	Soybean	Corn	Soybean	Corn	Soybean
Cultivar		2A401PW	M7110 IPRO	2B433PW	SYN1163RR	B2433PWU	SPEED	MG545 PWU	Bmx Foco IPRO	P3858PWU	Bmx Foco IPRO
Density		3 seeds m^−1^	14.4 seeds m^−1^	3 seeds m^−1^	15 seeds m^−1^	3 seeds m^−1^	20 seeds m^−1^	3 seeds m^−1^	16 seeds m^−1^	3 seeds m^−1^	14 seeds m^−1^
Fertilization	N-sowing	80 kg ha^−1^	0 kg ha^−1^	30 kg ha^−1^	0 kg ha^−1^	30 kg ha^−1^	0 kg ha^−1^	30 kg ha^−1^	15 kg ha^−1^	15 kg ha^−1^	15 kg ha^−1^
	P_2_O-sowing	80 kg ha^−1^	80 kg ha^−1^	80 kg ha^−1^	80 kg ha^−1^	80 kg ha^−1^	80 kg ha^−1^	80 kg ha^−1^	80 kg ha^−1^	80 kg ha^−1^	80 kg ha^−1^
	K_2_O-sowing	80 kg ha^−1^	80 kg ha^−1^	90 kg ha^−1^	80 kg ha^−1^	90 kg ha^−1^	80 kg ha^−1^	90 kg ha^−1^	80 kg ha^−1^	60 kg ha^−1^	80 kg ha^−1^
	N-sidedressing	70 kg ha^−1^		100 kg ha^−1^		100 kg ha^−1^		100 kg ha^−1^		100 kg ha^−1^	
	S-sidedressing	-		-		-		-		10 kg ha^−1^	
		Intercropped		Intercropped		Intercropped		Intercropped		Intercropped	
		Corn		Corn		Corn		Corn		Corn	
Cultivar		2A401PW		2B433PW		B2433PWU		MG 545 PWU		P3858PWU	
Density		3 seeds m^−1^		3 seeds m^−1^		3 seeds m^−1^		3 seeds m^−1^		3 seeds m^−1^	
Fertilization	N-sowing	80 kg ha^−1^		30 kg ha^−1^		30 kg ha^−1^		30 kg ha^−1^		15 kg ha^−1^	
	P_2_O-sowing	80 kg ha^−1^		80 kg ha^−1^		80 kg ha^−1^		80 kg ha^−1^		80 kg ha^−1^	
	K_2_O-sowing	80 kg ha^−1^		90 kg ha^−1^		90 kg ha^−1^		90 kg ha^−1^		60 kg ha^−1^	
	N-sidedressing	70 kg ha^−1^		100 kg ha^−1^		100 kg ha^−1^		100 kg ha^−1^		100 kg ha^−1^	
	S-sidedressing									10 kg ha^−1^	
		Brachiaria		Brachiaria		Brachiaria		Brachiaria		Brachiaria	
Cultivar		*U. ruziziensis*		*U. ruziziensis*		*U. ruziziensis*		*U. ruziziensis*		*U. ruziziensis*	
Density		10 kg ha^−1^		10 kg ha^−1^		10 kg ha^−1^		10 kg ha^−1^		8 kg ha^−1^	
Cultural value		51.80%		75.00%		75.00%		80.00%		85.00%	
		Millet		Millet		Millet		Millet		Millet	
Cultivar		ADR300		ADR300		ADR300		ADR300		BRS 1501	
Density		20 kg ha^−1^		20 kg ha^−1^		20 kg ha^−1^		20 kg ha^−1^		15 kg ha^−1^	
		Brachiaria		Brachiaria		Brachiaria		Brachiaria		Brachiaria	
Cultivar		*U. ruziziensis*		*U. ruziziensis*		*U. ruziziensis*		*U. ruziziensis*		*U. ruziziensis*	
Density		10 kg ha^−1^		10 kg ha^−1^		10 kg ha^−1^		10 kg ha^−1^		10 kg ha^−1^	
Cultural value		51.80%		75.00%		75.00%		80.00%		80.00%	

**Table A2:** Description of phytosanitary management for soybeans during the two years of evaluation for this work (2020/2021 and 2021/2022 crops).

Phytosanitary Management—Soybean 2020/2021		
Dose (c.p.)		Active Ingredient
	1st application	
200 mL ha^−1^ Wetcit^®^		Orange oil
2.5 L ha^−1^ Shadow Transorb^®^		Glyphosate
0.8 L ha^−1^ Viance^®^		Clethodim
	2nd application	
150 mL ha^−1^ Mustang 350 EC^®^		Zeta-cypermethrin
	3rd application	
300 mL ha^−1^ Galil SC^®^		Bifenthrin + Imidacloprid
300 mL ha^−1^ Approach Prima^®^		Cyproconazole + Picoxystrobin
200 mL ha^−1^ Wetcit^®^		Orange oil
	4th application	
200 mL ha^−1^ Wetcit^®^		Orange oil
300 mL ha^−1^ Approach Prima^®^		Cyproconazole + Picoxystrobin
1 L ha^−1^ Talismã^®^		Bifenthrin + Carbosulfan
